# Observations supporting hypothetical commensalism and competition between two *Campylobacter jejuni* strains colonizing the broiler chicken gut

**DOI:** 10.3389/fmicb.2022.1071175

**Published:** 2023-01-26

**Authors:** Sophie Chagneau, Marie-Lou Gaucher, William P. Thériault, Philippe Fravalo, Alexandre Thibodeau

**Affiliations:** ^1^Research Chair in Meat Safety, Department of Pathology and Microbiology, Faculté de Médecine Vétérinaire, Université de Montréal, Saint-Hyacinthe, QC, Canada; ^2^Swine and Poultry Infectious Diseases Research Center (CRIPA), Department of Pathology and Microbiology, Faculté de Médecine Vétérinaire, Université de Montréal, Saint-Hyacinthe, QC, Canada; ^3^Groupe de Recherche sur les Maladies Infectieuses en Production Animale (GREMIP), Department of Pathology and Microbiology, Faculté de Médecine Vétérinaire, Université de Montréal, Saint-Hyacinthe, QC, Canada; ^4^Chaire Agroalimentaire du Conservatoire National des Arts et Métiers, Paris, France

**Keywords:** *Campylobacter jejuni*, broiler chickens, gut colonization, extraintestinal dissemination, commensalism, competition, tight junction proteins

## Abstract

*Campylobacter jejuni* is the most prevalent bacterial foodborne pathogen in humans. Given the wide genetic diversity of *C. jejuni* strains found in poultry production, a better understanding of the relationships between these strains within chickens could lead to better control of this pathogen on farms. In this study, 14-day old broiler chickens were inoculated with two *C. jejuni* strains (10^3^ or 10^7^ CFU of D2008b and 10^3^ CFU of G2008b, alone or together) that were previously characterized *in vitro* and that showed an opposite potential to compete for gut colonization in broilers. Liver samples and ileal and cecal contents were collected and used to count total *C. jejuni* and to quantify the presence of each strain using a strain specific qPCR or PCR approach. Ileal tissue samples were also collected to analyze the relative expression level of tight junction proteins. While a 10^3^ CFU inoculum of D2008b alone was not sufficient to induce intestinal colonization, this strain benefited from the G2008b colonization for its establishment in the gut and its extraintestinal spread. When the inoculum of D2008b was increased to 10^7^ CFU – leading to its intestinal and hepatic colonization – a dominance of G2008b was measured in the gut and D2008b was found earlier in the liver for birds inoculated by both strains. In addition, a transcript level decrease of *JAM2*, *CLDN5* and *CLDN10* at 7 dpi and a transcript level increase of *ZO1*, *JAM2*, *OCLN*, *CLDN10* were observed at 21 dpi for groups of birds having livers contaminated by *C. jejuni*. These discoveries suggest that *C. jejuni* would alter the intestinal barrier function probably to facilitate the hepatic dissemination. By *in vitro* co-culture assay, a growth arrest of D2008b was observed in the presence of G2008b after 48 h of culture. Based on these results, commensalism and competition seem to occur between both *C. jejuni* strains, and the dynamics of *C. jejuni* intestinal colonization and liver spread in broilers appear to be strain dependent. Further *in vivo* experimentations should be conducted to elucidate the mechanisms of commensalism and competition between strains in order to develop adequate on-farm control strategies.

## 1. Introduction

*Campylobacter jejuni* is responsible for approximatively 90% of campylobacteriosis cases in humans (Kaakoush et al., 2015). Campylobacteriosis is a severe but self-limiting gastro-enteritis with an infectious dose as low as 500 bacteria (Robinson, 1981; Black et al., 1988) that lasts an average of 6 days. In some cases, it can lead to severe conditions such as the Guillain-Barré syndrome, Miller Fisher syndrome, reactive arthritis, irritable bowel syndrome, and celiac disease (Facciolà et al., 2017). In Canada, there was an annual incidence rate of 29.14 per 100,000 population of campylobacteriosis in 2018 (Public Health Agency of Canada, 2021), while in Europe, there were 220,682 human cases attributed to this pathogen in 2019, that was reported as the first cause of notified zoonosis (European Food Safety Authority and European Centre for Disease Prevention and Control, 2021). The principal sources of human contamination by *C. jejuni* are poultry, dairy products like unpasteurized milk, and water (Domingues et al., 2012). Poultry products are recognized as the primary source of exposure for humans, mainly due to the high *C. jejuni* loads that can be found in the intestines of poultry carriers (up to 10^9^ CFU/g of intestinal content; Greig and Ravel, 2009). Humans can be infected by the consumption of contaminated or cross-contaminated undercooked chicken products (Nadeau et al., 2002). The majority of campylobacteriosis cases in humans are sporadic, except for virulent *Campylobacter* species that would appear to be associated with diffuse outbreaks (Llarena et al., 2016). Some outbreaks are reported in the literature after the consumption of undercooked chicken meat (Yu et al., 2010; Llarena and Kivistö, 2020), chicken liver pâté (Little et al., 2010; Moffatt et al., 2016; Lanier et al., 2018), and unpasteurized milk (Centers for Disease Control and Prevention (CDC), 2002; Burakoff et al., 2018).

According to the season and country, up to 95% of broiler chicken flocks can be contaminated with *C. jejuni* by 2 weeks of age (Stern et al., 2001; Newell and Fearnley, 2003; van Gerwe et al., 2009). Absence of intestinal colonization by *C. jejuni* before 2 weeks of age could be partially due to the presence of maternal antibodies in younger birds (Sahin et al., 2003) and/or to intestinal microbiota properties (Laisney et al., 2004; Han et al., 2016). In birds, the infection occurs *via* the fecal-oral route, leading to a rapid spread of *C. jejuni* in the flock (Sahin et al., 2015). Once ingested, *C. jejuni* migrates to the chicken intestine and especially to the caeca; this migration is most probably promoted by colonization factors related to chemotaxis and motility (Hermans et al., 2011). After colonizing the gut, the bacterium can disseminate to internal organs such as the liver through the bloodstream (Cox et al., 2005, 2007; Richardson et al., 2011). According to the literature, an increase of the paracellular permeability, associated with alterations of the expression of tight junctions proteins could explain this phenomenon (Lamb-Rosteski et al., 2008; von Buchholz et al., 2022). At the slaughterhouse, sources of contamination are numerous: contaminated feathers, gut leakage caused by the defeathering process, evisceration, and finally cross-contamination *via* direct contact with various contaminated surfaces within the production environment (Newell et al., 2001). Moreover, *C. jejuni* extraintestinal spread may present an additional challenge as *C. jejuni* was detected inside of retail chicken livers, with a prevalence ranging from 10 to 100% and with bacterial loads varying from 10 to 10^5^ CFU/g of liver (Barot et al., 1983; Whyte et al., 2006; Noormohamed and Fakhr, 2012; Harrison et al., 2013; Berrang et al., 2018).

Usually, *C. jejuni* colonization is asymptomatic in chickens, which explains why this microorganism is often considered by some authors to be a commensal or commensal-like bacterium of the chicken gut. A few studies have observed in experimental settings that particular strains of *C. jejuni* have caused mild clinical signs in broiler chickens, mainly transient diarrhea, arthritis, and reduction in body weight gain (Ruiz-Palacios et al., 1981; Dhillon et al., 2006; Gharib Naseri et al., 2012; Awad et al., 2015; Alpigiani et al., 2017). Interestingly, one study isolated *C. jejuni* from the liver of broiler chickens affected with avian vibrionic hepatitis, but a causative link remained unclear (Jennings et al., 2011).

The wide genetic diversity of *C. jejuni* strains in poultry production has been revealed using molecular biology techniques (Wassenaar and Newell, 2000). Most of the studies have shown that multiple *C. jejuni* genotypes could be simultaneously isolated from the cecal content or feces of broiler chickens from the same flock (Thomas et al., 1997; Hiett et al., 2002; Messens et al., 2009), suggesting that birds can be colonized with different *C. jejuni* strains on the same farm. Among these multiple genotypes, the dominance of one or sometimes two *C. jejuni* strains in the cecal content or feces of broiler chickens within the same flock is noted most of the time, from the time of contamination until slaughter (Newell et al., 2001; Nadeau et al., 2002; Ring et al., 2005; Ellerbroek et al., 2010), and this has been reported to be due to the different abilities of these strains to colonize broiler chickens (Ringoir and Korolik, 2003; Chaloner et al., 2014; Pielsticker et al., 2016). Thus, when a multi-strain colonization occurs, the involved *C. jejuni* strains compete and some may become predominant, displacing the others, as it has been confirmed experimentally (Korolik et al., 1998; Konkel et al., 2007; Coward et al., 2008; Calderón-Gómez et al., 2009). In a previous study aimed at better understanding these different abilities to colonize the chicken gut, *C. jejuni* strains isolated from the caeca of broiler chickens were characterized *in vitro* and classified according to their autoagglutination, chemotaxis, adhesion, and invasion properties known to contribute to an effective gut colonization. It was also found that these strains showing different phenotypes possess different abilities to compete for gut colonization in birds (Thibodeau et al., 2015a).

As for extraintestinal spread, one or more genotypes of *C. jejuni* have been found inside retail chicken livers but only one genotype seemed to be dominant (Berrang et al., 2018, 2019). Experimentally, the strains’ ability to colonize the chicken intestine and to spread to organs, including the liver, was determined using a model where birds were inoculated with a dominant *C. jejuni* isolate (Jennings et al., 2011; Firlieyanti et al., 2016; Pielsticker et al., 2016). Despite successful intestinal colonization, hepatic spread was highly variable. Using animal experimentations, other authors also demonstrated that *C. jejuni* strains from human and avian origin would have different abilities to spread to the liver in broiler chickens (Chaloner et al., 2014; Pielsticker et al., 2016). Finally, in a recent study conducted at the slaughterhouse, the authors isolated a dominant strain of *C. jejuni* from the cecal content of broilers that was different from the one isolated from the inside of the liver of the same bird (Berrang et al., 2019). Thus, the extra-intestinal *C. jejuni* spread to the liver appears to also be strain specific and would involve other unknown factors and mechanisms that appear to be different from those governing intestinal colonization.

These new insights into the relationship between some *C. jejuni* strains inside the chicken intestinal environment and liver is therefore raising concerns that the presence of the pathogen in birds might impact animal and public health. However, how intestinal colonization and extraintestinal spread are linked or related, especially in the context of a multi-*C. jejuni* strain colonization, remains unclear.

The aim of this study was therefore to further analyze the intestinal co-colonization of two thoroughly characterized *C. jejuni* strains with an opposite potential to compete for gut colonization in broiler chickens. Using different ratios of these strains, we investigated the impact of this multi-strain inoculation on each strain, on cecal and ileal colonization of birds, on the extra-intestinal spread of each strain to the liver and on the expression of tight junction proteins from ileal tissue. We also explored the underlying mechanisms of the intestinal competition between the same two *C. jejuni* strains by *in vitro* co-culture assays.

## 2. Materials and methods

### 2.1. *Campylobacter jejuni* strain selection and culture conditions

Two *C. jejuni* strains, isolated from the caeca of commercial broiler chickens, were used in the current study. These strains, identified as G2008b and D2008b, have different comparative genetic fingerprinting profiles. These strains were previously characterized *in vitro* in our laboratory and identified as strong competitor (G2008b) and weak competitor (D2008b; Thibodeau et al., 2015a). For the inoculation of the birds in the *in vivo* trial, *C. jejuni* strains were grown on tryptic soy blood agar plates (Fisher Scientific, Ottawa, ON, Canada) for 24 h at 42°C in jars (2.5 l) under microaerobic conditions (80% N_2_, 10% CO_2_, 5% H_2_, and 5% O_2_) using the gas pack CampyGen system (Oxoid, Ottawa, ON, Canada) before being suspended in sterile tryptone salt solution (0.1% tryptone (w/v) and 0.85% NaCl (w/v), Fisher Scientific). An absorbance of 1.0 at 600 nm corresponding to about 10^9^ CFU/ml, was measured and bacterial suspensions were diluted to obtain the desired concentration. After oral gavage, inoculate were plated on tryptic soy blood agar plates to verify the correct dose.

The co-culture assays were realized after the animal phase in an attempt to confirm some observation made *in vivo*. For co-culture assays, both *C. jejuni* strains were grown on blood agar plates as described above. Bacteria were suspended separately in a sufficient volume of sterile tryptone salt solution to obtain an absorbance of 1.0 measured at 600 nm using a Novaspec II Spectrophotometer (Pharmacia Biotech, NJ, United States). Five milliliters of D2008b or 5 ml of G2008b were inoculated separately in 45 ml of Mueller-Hinton (MH) broth (BD, BBL, Franklin Lakes, NJ, United States), and 5 ml of D2008b and 5 ml of G2008b were inoculated together in 40 ml of MH broth in a 250 ml Erlenmeyer flask. Bacterial suspensions were incubated in a MaxQ 4000 orbital shaker (Fisher Scientific) at 150 rpm for 24 h at 42°C under microaerobic conditions (Oxoid), as previously described, corresponding to the pre-culture step. For each condition, 5 ml was then suspended in 45 ml of fresh MH broth in a 250 ml Erlenmeyer flask and were incubated in the MaxQ 4000 orbital shaker at 150 rpm for 72 h at 42°C in jars under microaerobic conditions as previously described (Oxoid). At *T* = 0, *T* = 24 h, *T* = 48 h, and *T* = 72 h, 1 ml of *C. jejuni* suspensions were used to measure absorbance at 600 nm, and 2 ml was collected and centrifuged at 15,000 × *g* for 5 min at 4°C (VWR, Mississauga, ON, Canada). Supernatants were removed and bacterial pellets were stored at −20°C until further analysis. MH broth without bacteria was used as negative control to verify the absence of contamination. Co-culture assays were performed in three independent replicates.

### 2.2. Animal experiments

The current *in vivo* study was conducted with the approval by the Comité d’Éthique sur l’Utilisation des Animaux (CÉUA) of the Faculté de Médecine Vétérinaire of the Université de Montréal (certificate number: 19-Rech-2039). A total of 197 one-day-old Ross 308 male broiler chickens were purchased from a local hatchery where they were vaccinated against Marek’s disease and infectious bronchitis. Chicks were carried to the Centre de Recherche Avicole of the Faculté de Médecine Vétérinaire, an animal facility suitable for experiments requiring level 2 biosecurity measures. Birds were divided randomly into two groups (Room 1 and Room 2), with an *ad-libitum* access to feed and water. Feed consisted of a standard mash commercial formulation (Supplementary Table S1). Birds were raised on wood-shavings that were not changed during the trial. In-house heating and lighting programs were applied. The birds were orally inoculated at 14 days old. Room 1 was divided into four groups: (1) not inoculated (control #1); (2) inoculated with 10^3^ CFU of D2008b (10^3^ D2008b); (3) inoculated with 10^3^ CFU of G2008b (10^3^ G2008b); and (4) inoculated with 10^3^ CFU of both strains at the same time (mix #1). In room 2, we decided to increase the inoculum of D2008b only – a weakly competitive strain – to explore its impact on competition with G2008b – a strongly competitive strain. Therefore, room 2 was also divided into four groups: (1) not inoculated (control #2); (2) inoculated with 10^7^ CFU of D2008b (10^7^ D2008b); (3) inoculated with 10^3^ CFU of G2008b (10^3^ G2008b); and (4) inoculated with 10^7^ CFU of D2008b and 10^3^ CFU of G2008b at the same time (mix #2). For both rooms, each group was housed in individual pens that were separated by pieces of Plexiglas to prevent contamination between groups. When there was a need to enter the pens, a clean new pair of boots was required to be put on right before stepping into the pens. Birds were visited daily. At 1 dpi (day post inoculation), 7 dpi, and 21 dpi, eight or nine birds per group were weighed and sedated with 0.8 ml/kg of a stock solution containing 50 ml of ketamine (100 mg/ml) and 12.5 ml of xylazine (100 mg/ml). Chickens were then euthanized by cervical dislocation. The same lobe of liver, distal ileum, and caeca were collected for each bird, kept on ice, and carried to the laboratory. The intestinal content and livers were used fresh for total *C. jejuni* counts. In cryotubes, about 1 g of intestinal contents were also frozen in liquid nitrogen and stored at −80°C until DNA extraction for strain specific qPCR. Prior to being processed for *C. jejuni* enumeration, livers were dipped in 70% ethanol for 5 s and the ethanol excess was burned-off to remove possible external contaminations. Moreover, ileal tissue samples of birds were freshly collected during necropsies, washed with RNase-free PBS (Invitrogen) and stored at −80°C in RNAlater Stabilization Solution (Invitrogen) until further analyzes.

### 2.3. Total *Campylobacter jejuni* counts

For each inoculated bird, 1 g of cecal and ileal content was serially diluted 10-fold in sterile tryptone salt solution. For uninoculated birds, 1 g of cecal and ileal contents were diluted 10-fold in sterile tryptone salt. Surface-sterilized liver lobes from inoculated and uninoculated birds were smashed to expose the internal tissue, resuspended 5-fold in sterile tryptone salt, and stomached for 1 min. One hundred microliters of each dilution were plated on Butzler agar plates (Oxoid) that were incubated in a microaerobic atmosphere (Oxoid) at 42°C for 48 h to enumerate total *C. jejuni*. A maximum of 10 isolated colonies from each contaminated liver were cultivated on blood agar plates and incubated under microaerobic atmosphere (Oxoid) at 42°C for 48 h. Each isolate was stored at −80°C in a freezing medium containing Brucella Broth (BD BBL) supplemented with 5% of sucrose (w/v), 20% of glycerol (v/v), 0.4% of ascorbic acid (w/v), and 0.12% of agar (w/v) until strain identification.

### 2.4. DNA extraction

#### 2.4.1. DNA extraction from cecal and ileal contents

In tubes containing 500 mg of 0.1 mm silica spheres (MP Biomedical, Solon, OH, United States), 200 mg of cecal content or 300 mg of ileal contents were weighed. Seven hundred microliters of lysis buffer (500 mM Tris–HCl pH 8, 100 mM EDTA pH 8, 100 mM NaCl, and 1% SDS) were added in tubes. A mechanical lysis was accomplished with a FastPrep-24 5G Instrument (MP Biomedical), using three runs of 60 s at 6 m/s with incubation for 5 min on ice between each run. Samples were heated for 20 min at 95°C then kept again on ice for 5 min. A centrifugation was performed at 18,000 × *g* for 15 min at 4°C (VWR) and supernatant was used for DNA purification by phenol/chloroform (Sigma-Aldrich, St. Louis, MO, United States) as previously described (Thibodeau et al., 2015b). DNA purity was confirmed by Nanodrop 1000 (Fisher, Ottawa, ON, Canada).

#### 2.4.2. DNA extraction from *Campylobacter jejuni* colonies of contaminated livers

Colonies isolated from contaminated livers were grown on blood agar plates (Fisher scientific) under microaerobic conditions (Oxoid) at 42°C for 48 h. Bacteria were collected in 1.5 ml tubes and 100 μl of 6% Chelex^®^ 100 Resin solution (Bio-Rad, Mississauga, ON, Canada) were added. Tubes were vortexed 10 s and heated at 55°C for 30 min. After this step, tubes were heated at 98°C for 15 min and centrifuged at 14,000 × *g* for 5 min at 4°C (VWR). Supernatants containing DNA were collected for quantification.

#### 2.4.3. DNA extraction from *Campylobacter jejuni* pellets from co-culture assays

DNA extractions from *C. jejuni* pellets were performed with a PowerLyser PowerSoil DNA Isolation Kit (QIAGEN, Toronto, ON, Canada) according to the manufacturer’s instructions. A FastPrep-24 5G Instrument (MP Biomedical) was used for the mechanic lysis step, consisting of two runs of 60 s at 6 m/s.

All DNA samples were quantified by DeNovix QFX Fluorometer using a Qubit dsDNA BR assay kit (Fisher Scientific) and stored at −80°C or − 20°C until further analysis.

### 2.5. Selection of strain specific genes

Both *C. jejuni* strains were grown overnight at 42°C on OMHA + blood plates under microaerobic conditions and genomic DNA was extracted using Epicentre Metagenomic DNA Isolation kits for Water (Illumina) according to the manufacturer’s instructions, as previously described (Clark et al., 2016). Briefly, quantification of DNA was performed by DeNovix QFX Fluorometer using Qubit dsDNA BR assay kit (Fisher Scientific). Sample libraries were prepared using a MiSeq Nextera^®^ XT DNA library preparation kit (Illumina). Whole genome sequencing was performed by 250 bp paired end read sequencing on the Illumina MiSeq sequencer using a MiSeq^®^ Reagent Kit V2 and 500 cycles on the Illumina MiSeq platform. Sequence reads were assembled into contigs using INNUca 2.6. Pangenomic annotations were performed by Roary (Supplementary Data S1). Raw reads can be accessed on NCBI under the reference number PRJNA903792. From pangenomic annotations, strain specificity, using the NCBI BLAST tool and conventional PCRs, was verified for many genes. Among these genes, two genes unique to G2008b (*Lps*A and *Dms*B) and two genes unique to D2008b (*Mcr*BC and *Rim*P) were selected as candidates for qPCR/PCR targets for the quantification/identification of each strain in the samples (Supplementary Figure S1).

### 2.6. Specific quantitative/conventional PCR for *Campylobacter jejuni* strains

Both strains were quantified from ileal and caecal contents as well as from co-culture assays using strain specific quantitative PCRs. *C. jejuni* strains were identified from colonies isolated from contaminated livers by strain specific conventional PCRs. The same primers (Table 1), designed using the NCBI Primer-BLAST tool, were used to perform the strain specific conventional and quantitative PCRs described below.

**Table 1:** Primer sequences used for strain specific PCR and qPCR.

Gene	Strain specificity	Primer sequences (5′-3′)	Amplicon sizes (bp)
*Lps*A	G2008b	F: TGCGAAACTTGGTTTATGGAAGG	118
		R: CCAATGCATCTTTTGGGCGA	
*Dms*B	G2008b	F: AGATGGAAGTTTTGAGCAAAGTGT	138
		R: TCATCGATTGCTACTATTCCCCATT	
*Mcr*BC	D2008b	F: TCGATGTCCGCATGCTTGT	123
		R: TCTTTCTCACCACCTCTTGTCTT	
*Rim*P	D2008b	F: TGTACAAAAAGAAGGCGGGGTA	142
		R: GCTAAGTTTTCTTTCAAGTCCTGGT	

For strain specific conventional PCRs, for each gene, 10 ng of DNA from colonies isolated from livers were amplified in a final reaction volume of 20 μl containing 500 nM of each primer (Invitrogen, Burlington, ON, Canada), 1X PCR Buffer (Biobasic, Markham, ON, Canada), 2 mM of MgSO4 (Biobasic), 0.2 μM of dNTPs (Biobasic), and 1 unit of Taq DNA Polymerase High Purity (Biobasic). The PCR program consisted of an initial denaturation step of 10 min at 95°C followed by 35 cycles of 10 s at 95°C, 10 s at 60°C, 10 s at 72°C, and a final elongation step of 1 min at 72°C. Conventional PCRs were performed using a Mastercycler^®^ nexus thermocycler (Eppendorf Canada, Mississauga, ON, Canada). The PCR products were analyzed by electrophoresis in a 2% agarose gel containing 0.01% SYBR Safe DNA gel stain (Invitrogen).

For strain specific quantitative PCRs, 10 ng of DNA were amplified in a final reaction volume of 20 μl containing 500 nM of each primer (Invitrogen) and 1X Master Mix EvaGreen (Montréal Biotech Inc., Montréal, QC, Canada) for each gene. This PCR program described above was used with a final step consisting of a high-resolution melting analysis to verify the specificity of PCR products. Quantitative PCRs were performed using a Roche LightCycler^®^ 96 Real Time PCR thermocycler (Roche Canada, Laval, QC, Canada). The abundance of each specific gene was determined using a standard curve that was prepared by the following method. DNA from both *C. jejuni* strains were amplified with a strain specific conventional PCR as described. The specificity of each PCR was confirmed in 2% agarose gels. The PCR product concentration was measured by a DeNovix QFX Fluorometer using a Qubit dsDNA BR assay kit (Fisher Scientific) to determine the number of gene copies for each gene. PCR products were serially diluted 10-fold to obtain a standard curve from 10^8^ to 10^2^ gene copies per microliter. For all samples and standard curves, quantitative PCRs were performed in duplicate for each gene. The proportion of each *C. jejuni* strain in cecal content, ileal content, and *C. jejuni* pellets from co-culture assays was determined by averaging the abundance of *Lps*A and *Dms*B copy numbers for G2008b and the abundance of *Mcr*BC and *Rim*P for D2008b for each sample. According to the weight of cecal content, ileal content, or the volume of *C. jejuni* suspension from co-culture assays used for DNA extractions, the amount of *C. jejuni* strains were expressed in number of gene copies/g of intestinal content or in number of gene copies/mL of MH broth.

### 2.7. RNA extraction of ileal tissue samples and reverse transcription

Approximately 20 mg of ileum samples stored in RNAlater Stabilization Solution were rinsed twice with RNase-free PBS (Invitrogen). Tissues samples were put in tubes containing 700 mg of 1.4 mm ceramic spheres (MP Biomedical) and 1 ml of TRIzol Reagent (Invitrogen). A FastPrep-24 5G Instrument (MP Biomedical) was used for the mechanic lysis step, consisting of two runs of 30 s at 4 m/s with incubation for 5 min on ice between each run. Tubes were incubated at room temperature for 5 min. A centrifugation was performed at 12,000 × *g* for 10 min at 4°C and the supernatant was collected to perform RNA isolation according to the manufacturer’s protocol from TRIzol Reagent. RNA purity was confirmed by Nanodrop 1000 (Fisher). RNA was quantified by DeNovix QFX Fluorometer using a Qubit RNA BR assay kit (ThermoFisher Scientific). One microgram of RNA was used for the reverse transcription to cDNA using SuperScript IV VILO Master Mix (Invitrogen) according to the manufacturer’s instructions.

### 2.8. Real-time quantitative PCR (qRT-PCR) of cDNA

For each gene and bird, 1 μl of cDNA was amplified in duplicate in a final reaction volume of 10 μl containing 500 nM of each primer (Invitrogen), 1X PowerTrack SYBR Green Master Mix (ThermoFisher Scientific) and 1X Yellow Sample Buffer (ThermoFisher Scientific). The PCR program consisted of an initial denaturation step of 10 min at 95°C followed by 30 cycles of 10 s at 95°C, 10 s at an annealing temperature indicated in Table 2 and a final step constituting a high-resolution melting to verify the specificity of PCR products. Quantitative PCRs were performed using a Roche LightCycler^®^ 96 Real Time PCR thermocycler (Roche Canada). Primers efficiency was considered adequate when it was between 90 and 110%. Due to their stable transcript level in ileal tissue in presence of *C. jejuni*, *β-actin* and *RPL32* were used as reference genes (Bagés et al., 2015). Relative transcript levels of target genes (*ZO1*, *JAM2*, *OCLN*, *CLDN5*, *CLDN10*) for inoculated birds were normalized to those of two reference genes and to the mean value of the control group for each room and time point using the 2^−ΔΔCt^ formula (Livak and Schmittgen, 2001).

**Table 2:** Primer sequences used for RT-qPCR.

Target gene	Primer sequences (5′-3′)	Annealing temperature	Reference
*β-actin*	F: CAACACAGTGCTGTCTGGTGGTA	60	St. Paul et al. (2011)
	R: ATCGTACTCCTGCTTGCTGATCC		
*Ribosomal protein L32 (RPL32)*	F: ATGGGAGCAACAAGAAGACG	58	Bagés et al. (2015)
	R: TTGGAAGACACGTTGTGAGC		
*Zonula occludens 1 (ZO1)*	F: CGTTCACGATCTCCTGAC	55	von Buchholz et al. (2021)
	R: CTGGTTTAGTTACCCTTTCATC		
*Junction-adhesion molecule 2 (JAM2)*	F: AGACAGGAACAGGCAGTGCT	60	Barekatain et al. (2019)
	R: TCCAATCCCATTTGAGGCTA		
*Occludin (OCLN)*	F: GTCTGTGGGTTCCTCATC	53	von Buchholz et al. (2021)
	R: CCAGTAGATGTTGGCTTTG		
*Claudin 5 (CLDN5)*	F: GCAGGCAAATAACTGCTTGGA	60	von Buchholz et al. (2021)
	R: AAAGTCTCAAAGGCGCACAG		
*Claudin 10 (CLDN10)*	F: TCCAACTGCAAGGACTTCCC	60	von Buchholz et al. (2021)
	R: GCCAAAGAAACCCAGACAGAC		

### 2.9. Statistical analyzes

Figures and statistical analyzes were performed with GraphPad Prism 9.2.0 (GraphPad Software Inc., La Jolla, CA). A Shapiro–Wilk test was used to verify data normality. To analyze statistical differences between groups according to the time point and room for the *in vivo* trial and co-culture assays, the specific statistical tests used are indicated in the figure legends. The limit of detection was set to 10^2^ CFU/g of cecal or ileal content for total *C. jejuni* counts and to 10^4^ gene copies/g of cecal or ileal content for qPCR results. Thus, in absence of detection, 9 × 10^1^ CFU/g of cecal or ileal contents were used to compare total *C. jejuni* counts and 9 × 10^3^ CFU/g to compare qPCR results between colonized chicken groups. Differences were statistically different when *p < 0.05, **p < 0.01, ***p < 0.001.

## 3. Results

### 3.1. General bird health

No hepatic gross lesions were observed at the time of necropsy and no significant difference in body weight was noted at 1, 7, and 21 days post infection (dpi; data not shown). Nevertheless, a transient diarrhea was noted at 1 dpi for 24 h only for birds infected simultaneously by both *C. jejuni* strains independently of the ratio of the two strains.

### 3.2. Cecal colonization of *Campylobacter jejuni* strains

**Figure 1:**
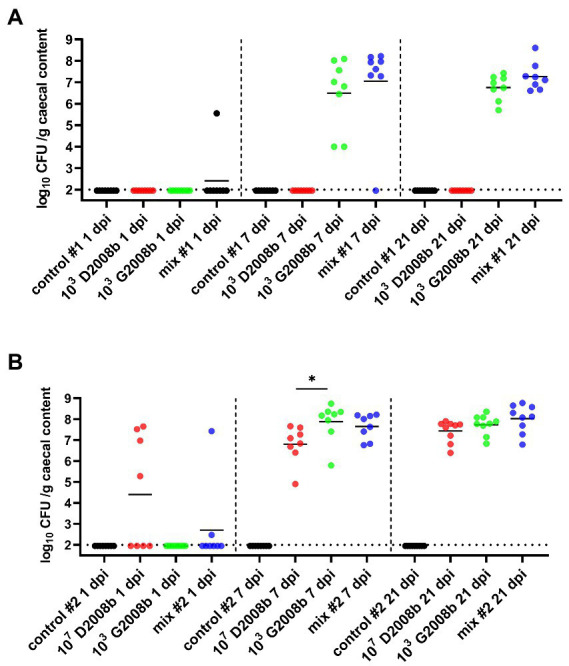
Total *Campylobacter jejuni* counts in cecal content (log_10_ CFU/g of cecal content) from birds at 1, 7, and 21 dpi for room 1 **(A)** and room 2 **(B)**. Dots correspond to individual birds. Horizontal bars represent median values, and horizontal dotted lines denote the limit of detection (2 log_10_ CFU/g of cecal content). Due to the low number of birds colonized at 1 dpi, statistical analyses were not performed. At 7 dpi and 21 dpi, statistical analyzes were conducted using a Mann–Whitney test **(A)** and a Kruskal-Wallis test followed by Dunn’s post-hoc tests with Bonferroni adjustments **(B)** to compare median values between colonized groups according to the time point and room. * indicates *p*<0.05.

Total *C. jejuni* counts were analyzed from the cecal content of all chickens by bacterial culture at 1, 7, and 21 dpi (Figure 1). In rooms 1 (same proportion of both strains) and 2 (higher ratio of D2008b), the absence of *C. jejuni* counts in the caeca from control birds was confirmed for each time point. At 1 dpi, only one bird and six birds were colonized by *C. jejuni* in ceca in room 1 and 2, respectively. Unlike birds inoculated with 10^3^ CFU of G2008b and both strains in room 1, the absence of *C. jejuni* from the caeca of birds inoculated with 10^3^ CFU of D2008b was noted at 7 and 21 dpi. At 7 and 21 dpi, no significant difference was observed for the total *C. jejuni* counts between birds inoculated with 10^3^ CFU of G2008b and with both strains. At 7 dpi, the number of *C. jejuni* in the cecal content of birds inoculated with G2008b was significantly higher (8.21 log_10_ CFU/g) than those from birds inoculated with D2008b (6.97 log_10_ CFU/g; *p* < 0.05) in room 2. At 21 dpi, no significant difference was observed between these two groups.

**Figure 2:**
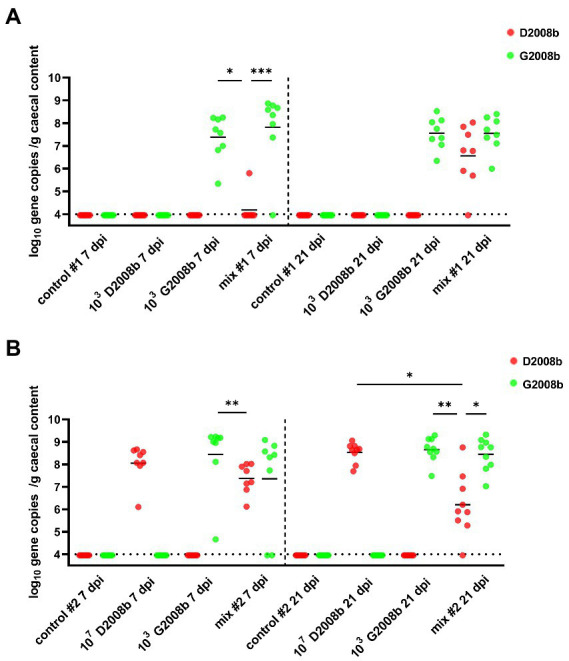
Amounts of *Campylobacter jejuni* strains in cecal content (log_10_ gene copies/g of cecal content) from birds at 7 and 21 dpi for room 1 **(A)** and room 2 **(B)**. Red and green dots correspond to the amount of D2008b and G2008b, respectively, for individual birds. Horizontal bars represent median values, and horizontal dotted lines denote the detection limit (4 log_10_ CFU/g of cecal content). Statistical analyzes were conducted using a Kruskal-Wallis test followed by Dunn’s post-hoc tests with Bonferroni adjustments to compare median values between colonized groups according to the time point and room. *, ** and *** indicate *p*<0.05, *p*<0.01 and *p*<0.001 respectively.

To assess the load of each *C. jejuni* strain in the cecal content, particularly in co-inoculation cases, strain specific qPCRs were performed (Figure 2). Strain specific genes *Mcr*BC and *Rim*P were targeted by qPCR to evaluate the D2008b load, while *Lps*A and *Dms*B were targeted for the G2008b load. Due to a low number of birds colonized at 1 dpi, qPCRs were only performed on samples collected at 7 and 21 dpi. For both time points, no strain specific genes were detected from the cecal content of birds infected with D2008b alone in room 1. For samples collected from this same room, only the G2008b specific genes were detected at 7 and 21 dpi in the cecal content of birds inoculated with G2008b. For birds inoculated with both *C. jejuni* strains, D2008b specific genes were detected from one bird at 7 dpi, while the other birds revealed the presence of G2008b solely. At 21 dpi, an increase in D2008b levels close to the G2008b levels was noticed for birds that had been co-inoculated, with the two strains reaching similar levels in the caeca. In room 2, at 7 and 21 dpi, only G2008b or D2008b specific genes were detected from the cecal content of birds inoculated with these single strains alone, respectively. For birds inoculated with both strains, similar levels of strain specific genes were observed in the cecal content at 7 dpi. At 21 dpi, a decrease in the abundance of D2008b was observed in co-inoculated birds. The number of gene copies of D2008b in the cecal content of co-inoculated birds (5.91 log_10_ gene copies/g) was significantly lower than the number found in birds inoculated with D2008b alone (8.65 log_10_ gene copies/g; *p* < 0.05). In addition, from the cecal content of broiler chickens inoculated with both strains, a 2.85 log_10_ difference was noted between the number of gene copies of D2008b and the number of gene copies of G2008b (*p* < 0.05).

### 3.3. Ileal colonization of *Campylobacter jejuni* strains

**Figure 3:**
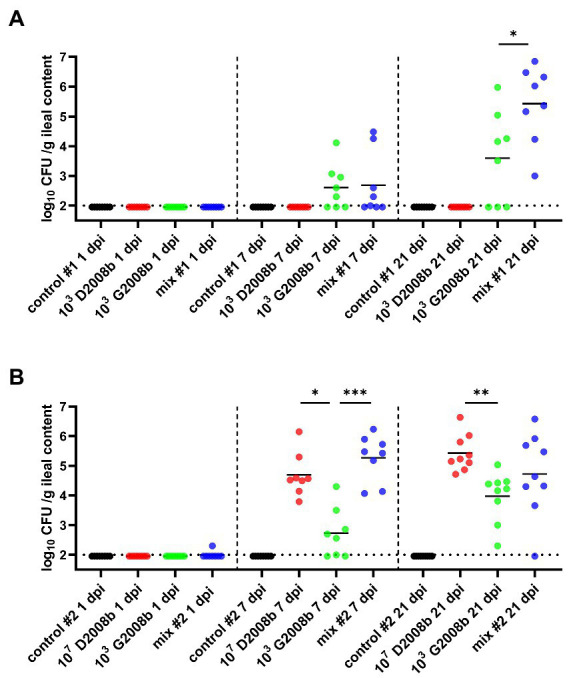
Total *Campylobacter jejuni* counts in ileal contents (log_10_ CFU/g of ileal content) from birds at 1, 7, and 21 dpi for room 1 **(A)** and room 2 **(B)**. Dots correspond to individual birds. Horizontal bars represent median values, and horizontal dotted lines denote the threshold (2 log_10_ CFU/g of ileal content). Due to the low number of birds colonized at 1 dpi, statistical analyzes were not performed. At 7 and 21 dpi, statistical analyzes were conducted using a Mann–Whitney test **(A)** and a Kruskal-Wallis test followed by Dunn’s post-hoc tests with Bonferroni adjustments **(B)** to compare median values between colonized groups according to the time point and room. *, ** and *** indicate *p*<0.05, *p*<0.01 and *p*<0.001 respectively.

Total *C. jejuni* counts were also analyzed from the ileal content of birds at 1, 7, and 21 dpi (Figure 3). For both rooms, absence of *C. jejuni* counts in the ileum from control birds was confirmed for each time point. At 1 dpi, only one bird was colonized by *C. jejuni* in ileum of birds in room 2. In room 1, the ileal content of birds inoculated with D2008b did not reveal the presence of *C. jejuni* at 7 and 21 dpi. At 7 dpi, total *C. jejuni* counts in the ileum of birds inoculated with G2008b and birds receiving both strains were very similar (2.45 log_10_ CFU/g and 2.15 log_10_ CFU/g respectively). At 21 dpi, the number of *C. jejuni* in the ileal content of co-inoculated chickens (5.69 log_10_ CFU/g) was significantly higher than those found in the ileum of birds inoculated with G2008b (3.84 log_10_ CFU/g; *p* < 0.05). In room 2 at 7 dpi, total *C. jejuni* counts in the ileal content of birds inoculated with D2008b and with both strains were higher by 1.91 log_10_ (*p* < 0.05) and 2.82 log_10_ (*p* < 0.001), respectively, in comparison to the counts observed for birds inoculated with G2008b. At 21 dpi, a 1.60 log_10_ increase in the number of *C. jejuni* in the ileal content of birds inoculated with G2008b was observed. Nevertheless, the number of *C. jejuni* found in the ileal content of chickens inoculated with G2008b was still lower by 1.00 log_10_ (*p* < 0.01) in comparison to the numbers found in the ileums of birds inoculated with D2008b.

**Figure 4:**
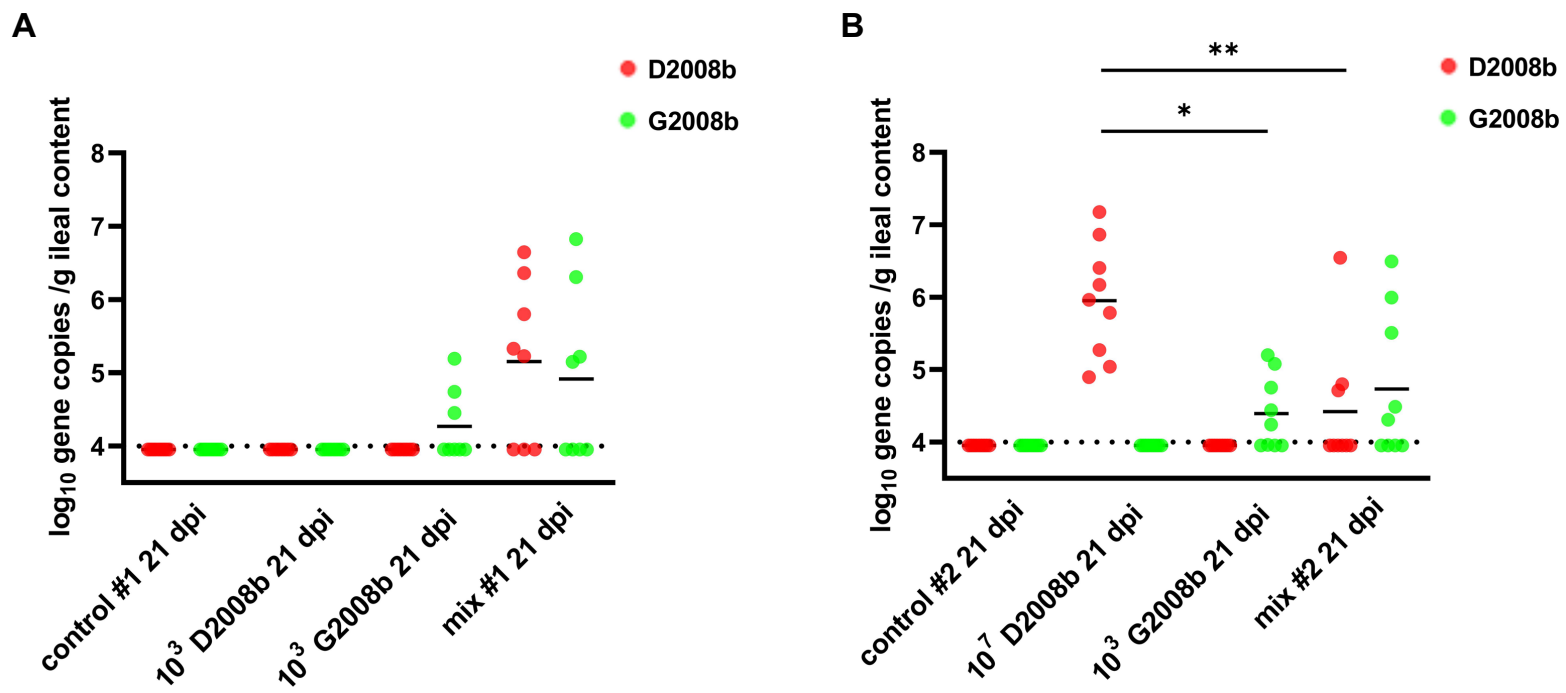
Amounts of *Campylobacter jejuni* strains in ileal content (log_10_ gene copies/g of ileal content) from birds at 7 and 21 dpi for room 1 **(A)** and room 2 **(B)**. Red and green dots correspond to the amount of D2008b and G2008b, respectively, for individual birds. Horizontal bars represent median values, and horizontal dotted lines denote the threshold (4 log_10_ CFU/g of ileal content). Statistical analyzes were conducted using a Kruskal-Wallis test followed by Dunn’s post-hoc tests with Bonferroni adjustments to compare median values between colonized groups according to the time point and room. * and ** indicate *p*<0.05 and *p*<0.01 respectively.

To assess the amount of each *C. jejuni* strain in the ileal content, strain specific qPCRs were performed (Figure 4). Strain specific genes *Mcr*BC and *Rim*P were targeted by qPCR to evaluate the D2008b load, while *Lps*A and *Dms*B were targeted for the G2008b load. Due to a low number of birds colonized at 1 dpi and to the low counts of *C. jejuni* enumerated by bacterial culture at 7 dpi, qPCRs were only performed on samples collected at 21 dpi. No strain specific gene was identified in the ileal content of chickens inoculated with D2008b in room 1. Only G2008b specific genes were detected in the ileal content of chickens inoculated with G2008b but close amounts of both strains were quantified in the ileal content of co-inoculated birds. At 21 dpi, only G2008b or D2008b specific genes were detected from the ileal content of birds inoculated with a single strain G2008b or D2008b, respectively, in room 2. From the ileal content of birds inoculated with D2008b, significantly higher numbers of D2008b gene copies (5.97 log_10_ gene copies/g) were detected compared to the number of G2008b gene copies found in birds inoculated with G2008b (4.24 log_10_ gene copies/g; *p* < 0.05) and to the number of D2008b gene copies found in birds receiving both strains (3.95 log_10_ gene copies/g; *p* < 0.01). No significant difference was noticed in the number of gene copies for D2008b and for G2008b in the ileal content of birds inoculated with both strains.

### 3.4. Extra-intestinal spread of *Campylobacter jejuni* strains to the liver

To evaluate the extra-intestinal spread to the liver of *C. jejuni* strains, total *C. jejuni* counts were determined at 1, 7, and 21 dpi by bacterial culture (Table 3). In both rooms, *C. jejuni* was not detected inside the liver of control birds and of birds inoculated with G2008b alone. In room 2, a significant extra-intestinal spread (>5 × 10^3^ CFU/g) of *C. jejuni* to the liver of birds inoculated with both strains was noticed at 7 dpi for seven of eight birds and to a lesser extent at 21 dpi for four of nine birds. However, in the liver of chickens inoculated with D2008b alone, the presence of *C. jejuni* was observed at 21 dpi only. Given that bacterial loads found in the liver were below the qPCRs threshold, bacterial DNA was extracted from *C. jejuni* colonies and specific conventional PCRs were performed to assess the identity of the isolates. Only D2008b specific genes (*Rim*P and *Mcr*BC) were detected for isolates recovered from the colonized livers (Table 4).

**Table 3:** Total *Campylobacter jejuni* counts in liver (CFU/g of liver) from birds at 1 dpi, 7 dpi and 21 dpi for rooms 1 and 2.

Chicken groups	Range of CFU/g liver (contaminated livers/birds per group per time point)		
	1 dpi	7 dpi	21 dpi
Control #1	(0/8)	(0/8)	(0/9)
10^3^ D2008b	(0/8)	(0/8)	(0/8)
103 G2008b	(0/8)	(0/8)	(0/8)
mix #1	(0/8)	(0/8)	1.36 × 10^2^–4.18 × 10^3^ (5/8)
Control #2	(0/8)	(0/8)	(0/9)
10^7^ D2008b	(0/8)	(0/8)	5.0 × 10^1^–4.73 × 10^3^ (5/9)
10^3^ G2008b	(0/8)	(0/8)	(0/9)
mix #2	(0/8)	> 5.0 × 10^3^ (7/8)	5.0 × 10^1^–4.41 × 10^3^ (4/9)

The limit of detection was 5.0 × 10^1^ CFU/g of liver.

**Table 4:** Identification of *Campylobacter jejuni* strains from contaminated livers by strain specific PCR.

Contaminated livers	G2008b specific genes		D2008b specific genes	
	*Lps*A	*Dms*B	*Mcr*BC	*Rim*P
mix #2 (7 dpi)	0/7	0/7	7/7	7/7
mix #1 (21 dpi)	0/5	0/5	5/5	5/5
10^7^ D2008b (21 dpi)	0/5	0/5	5/5	5/5
mix #2 (21 dpi)	0/4	0/4	4/4	4/4

### 3.5. Relative transcript level of tight junction proteins in ileum

**Figure 5:**
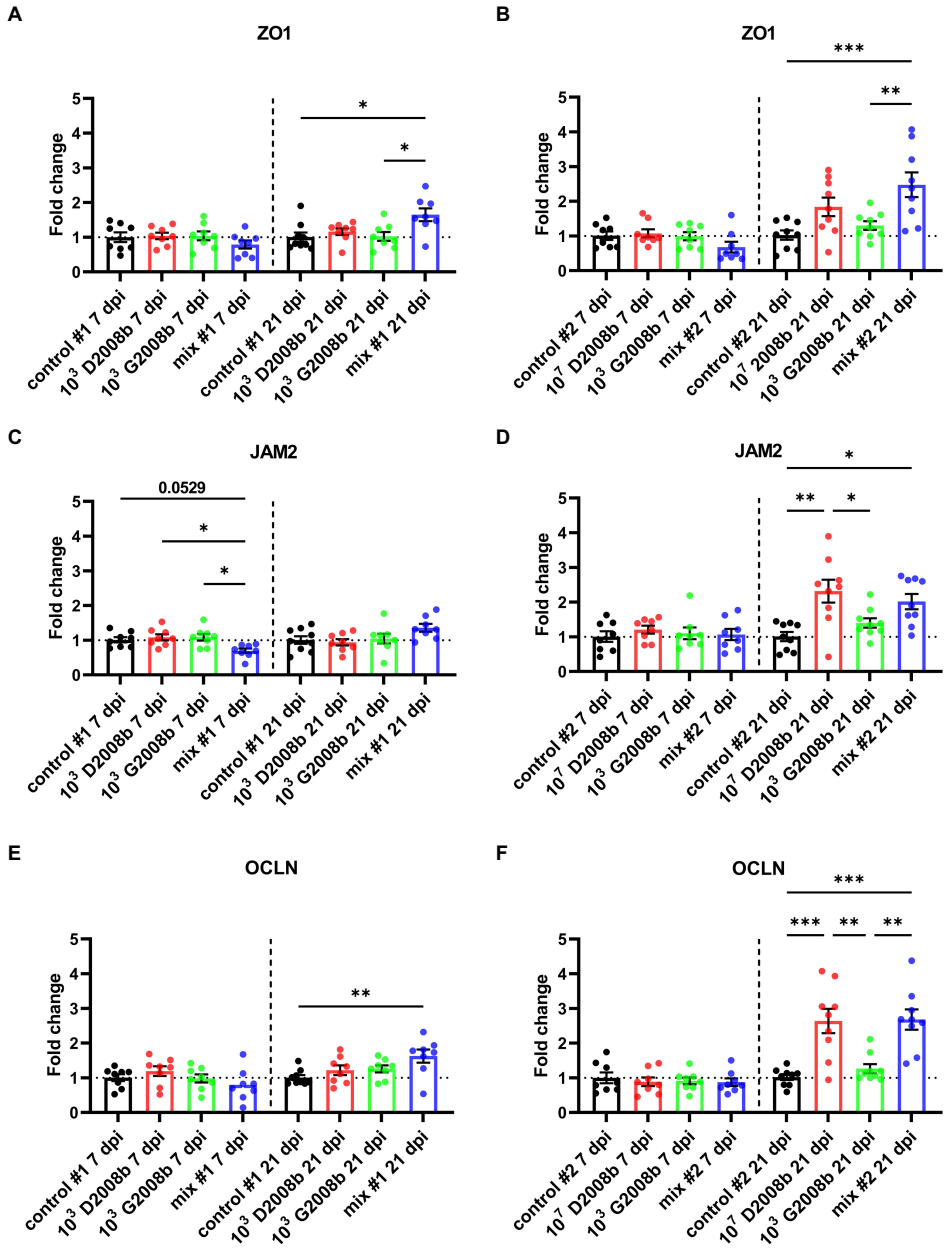
Relative gene expression of *ZO1* **(A,B)**, *JAM2* **(C,D)** and *OCLN* **(E,F)** in ileal tissue for rooms 1 and 2, respectively, at 7 dpi and 21 dpi. Dots correspond to the protein expression level normalized to two reference genes, *RPL32* and *β-actin*, and to the mean value of the control group. Vertical bars represent mean values ± SEM. ANOVA test followed by Tukey’s multiple comparisons tests were used to analyze statistical differences between chicken groups within the same time point. *, ** and *** indicate *p*<0.05, *p*<0.01 and *p*<0.001 respectively.

**Figure 6:**
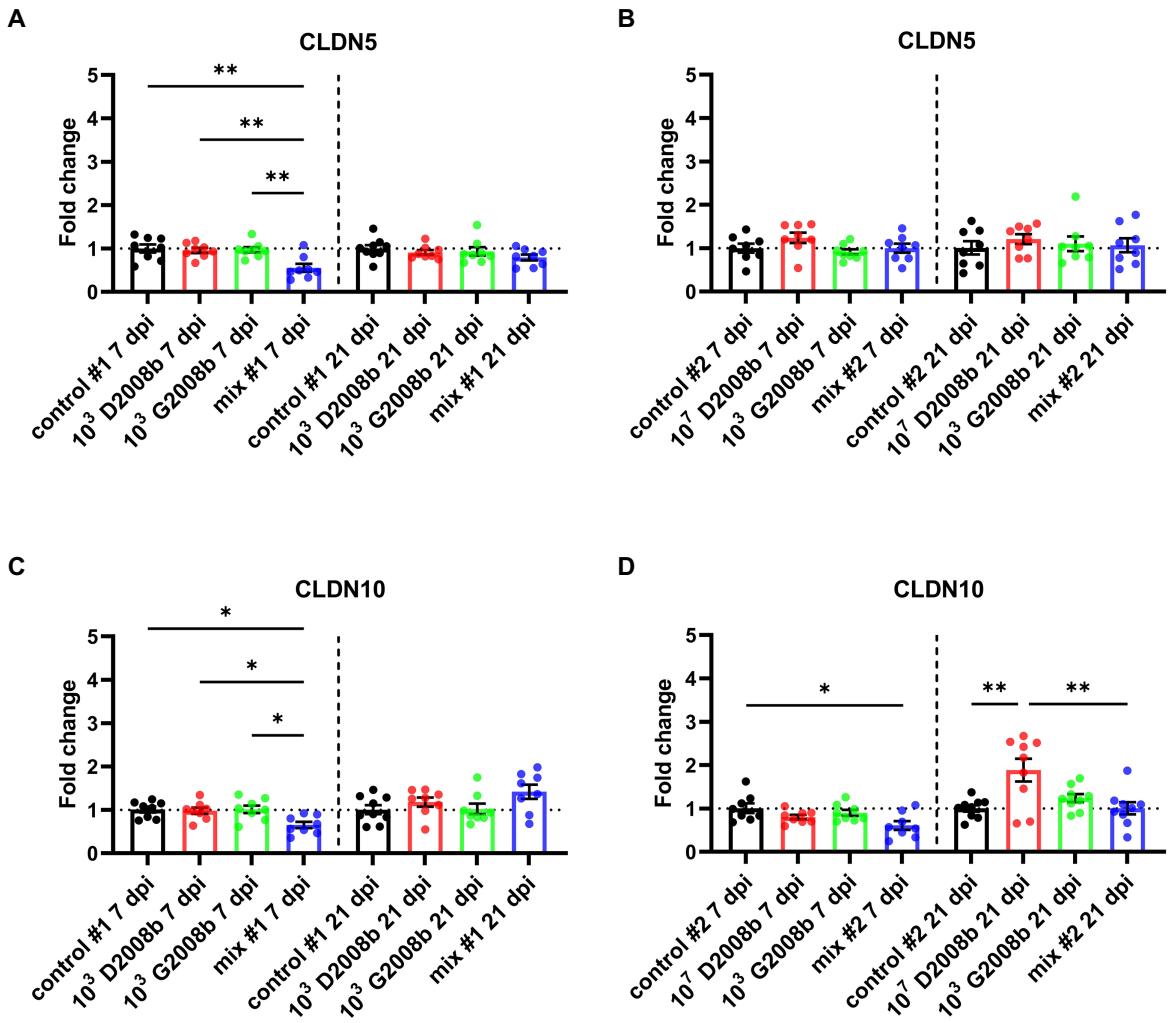
Relative gene expression of *CLDN5* **(A,B)** and *CLDN10* **(C,D)** in ileal tissue for rooms 1 and 2, respectively, at 7 dpi and 21 dpi. Dots correspond to the protein expression level normalized to two reference genes, *RPL32* and *β-actin*, and to the mean value of the control group. Vertical bars represent mean values ± SEM. ANOVA test followed by Tukey’s multiple comparison tests were used to analyze statistical differences between chicken groups within the same time point. * and ** indicate *p*<0.05 and *p*<0.01 respectively.

To evaluate the *C. jejuni* inoculation effect on relative transcript level of different tight junction proteins, RT-qPCR of *ZO1*, *JAM2*, *OCLN*, *CLDN5* and *CLDN10* were performed in ileal tissue samples from birds at 7 dpi and 21 dpi (Figures 5, 6). At 7 dpi, a significant decrease of the transcript level of *JAM2* and *CLDN5* was noted in ileal tissue from birds inoculated with both strains in room 1, and *CLDN10* from birds inoculated with both strains in both rooms. In contrast, at 21 dpi, a significant upregulation of the transcript level of *ZO1* and *OCLN* was observed in ileal tissue from birds inoculated with both strains in room 1; of *JAM2*, *OCLN* and *CLDN10* from birds inoculated with 10^7^ CFU of D2008b; of *JAM2* and *OCLN* from birds inoculated with both strains in room 2.

### 3.6. *In vitro* co-culture of *Campylobacter jejuni* strains

**Figure 7:**
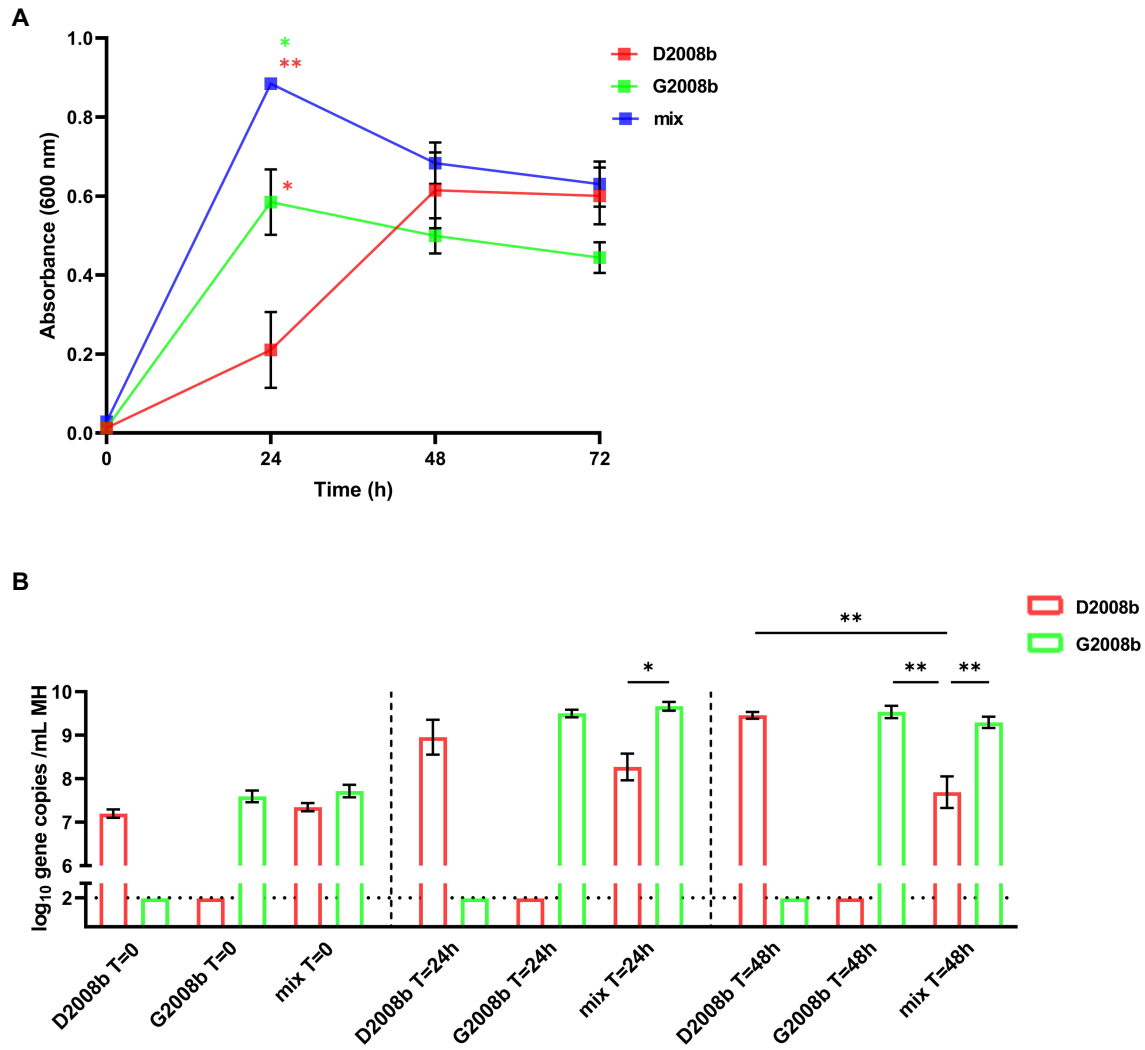
*In vitro* co-culture of both *Campylobacter jejuni* strains in MH broth. Both *C. jejuni* strains were grown separately and together in MH broth and absorbances at 600 nm were measured at different time points **(A)**. To evaluate the amounts of each strain, strain specific qPCRs were performed from *C. jejuni* pellets **(B)**. Vertical bars represent mean values ± SEM, and horizontal dotted lines denote the threshold (2 log_10_ gene copies/mL of MH broth). Student *t* tests were used to analyze statistical differences of absorbance and ANOVA test followed by Tukey’s multiple comparison tests to compare qPCR results by time point between conditions. Co-culture assays were conducted in three independent replicates. * and ** indicate *p*<0.05 and *p*<0.01 respectively.

Both *C. jejuni* strains were cultivated alone and together in MH broth in three independent culture assays, and absorbances were measured at different time points (Figure 7A). After 24 h, the absorbance of D2008b culture was significantly lower than that of G2008b (*p* < 0.05). Additionally, we observed that the absorbance of the co-culture broth was significantly higher than that of the single strain culture broths [G2008b (*p* < 0.05) and D2008b (*p* < 0.01)]. No significant difference was observed between conditions when co-cultures were extended to 48 h and 72 h. Quantitative PCRs were performed to evaluate the relative amount of each *C. jejuni* strain specific gene in bacterial co-culture and single strain cultures (Figure 7B). At *T* = 0, no significant difference was observed between conditions. At *T* = 24 h and *T* = 48 h, no significant difference between single strain cultures was observed. However, at *T* = 48 h, the number of D2008b gene copies was lower in the presence of G2008b (*p* < 0.01).

## 4. Discussion

Most *in vivo* experiments in broilers available in the literature have been conducted using a single *C. jejuni* strain. However, considering the wide variety of *C. jejuni* strains observed at the broiler farm level, the current study evaluates the impact of the presence of two distinct *C. jejuni* strains – alone and together – on the intestinal colonization and hepatic spread of the bacterium in broiler chickens to approximate the reality on farms more closely.

Although uninoculated birds were raised in the same room as inoculated birds, they were not colonized by *C. jejuni* throughout the animal experiments. Moreover, birds inoculated with a single *C. jejuni* strain were shown to have been colonized only by that strain. All measures taken in the animal facility to prevent contamination between groups were very effective, thereby to compare the intestinal colonization level, the hepatic spread of *C. jejuni* and the transcript level of tight junctions for birds inoculated by single or both strain(s) without room effect.

In both rooms, few birds were colonized in caeca at 1 day post infection (dpi), and even fewer birds were found to be colonized in ileum after analysis, due especially to the fact that the dose and inoculated strain(s) would have had an influence on early intestinal colonization. It has already been described that, depending on the strain, the cecal colonization of broilers could start at 1 dpi or later, and that all birds were colonized between 3 dpi and 14 dpi (Young et al., 1999; Knudsen et al., 2006). In this study, at 7 and 21 dpi, broiler chickens inoculated with 10^3^ CFU of D2008b alone were never shown to be colonized throughout the experiment, unlike birds inoculated with 10^3^ CFU of G2008b alone, with 10^7^ CFU of D2008b, or with both strains. Although both strains were originally isolated from the caeca of broiler chickens, it seems that a 10^3^ CFU inoculum of D2008b was not sufficient to induce colonization and the minimum dose of D2008b required for an effective intestinal colonization appears to be higher for this strain compared to G2008b. It has already been shown that a minimum dose of 10^2^ to 10^4^ CFU per bird, according to the *C. jejuni* strain used, is required for effective colonization in 14-day-old chickens (Ringoir and Korolik, 2003; Dhillon et al., 2006; Knudsen et al., 2006). These variations between strains are likely due to differences in phenotypic properties such as autoagglutination, chemotaxis, adhesion, and invasion, as previously reported (Thibodeau et al., 2015a). Although similar cecal colonization levels were observed between birds inoculated with 10^7^ CFU of the D2008b and those inoculated with 10^3^ CFU of G2008b at 21 dpi, D2008b appears to be better able to colonize the ileum. We suspect that the inoculum difference could impact colonization levels in the ileum or that the colonization dynamics of these two *C. jejuni* strains at the caeca and ileum levels could be different. Interestingly, our observations support similar observations reported by in broiler chickens inoculated with a same inoculum of two *C. jejuni* reference strains separately: M1 or 13126 (Chaloner et al., 2014). These authors showed that M1 preferentially colonized the caeca, whereas 13126 colonized the distal ileum at a higher level. The authors suggested that these strains would have different metabolic capacity and that variations throughout the gastrointestinal tract, including pH, oxygen tension, levels of bile, mucins, or even differences in the intestinal microbiota could explain these findings.

For birds housed in room 1 inoculated with both strains, D2008b seemed to benefit from the presence of G2008b for its establishment in the caeca and in the ileum as both strains were found at similar average levels at 21 dpi; D2008b alone did not colonize the birds. As the presence of D2008b did not affect the colonization levels of G2008b in the caeca and in the ileum, we suggest that a commensalism could occur between both *C. jejuni* strains at the intestinal level. Commonly, the term commensalism is used to define an interaction between two organisms that is beneficial for one without negatively or positively impacting the other. On the contrary, no commensalism was observed for the *in vitro* co-culture assays. Consequently, we think that direct interactions between strains would not underlie this proposed relationship observed *in vivo*. It was also demonstrated that other bacterial species can enhance *Campylobacter* colonization in mice (Wang et al., 2018) and in chickens (Yan et al., 2021), such as *Clostridium*. Therefore, investigation on the microbiota of the study animals is mandatory to see if different bacterial populations are associated to the different inoculated strains. When the inoculum of D2008b was increased by 4 log_10_ for birds housed in room 2, similar cecal colonization levels of D2008b were observed at 7 dpi, whether or not birds were inoculated with G2008b. However, we noted an average decrease of about 3 log_10_ in the colonization levels of D2008b in the presence of G2008b in caeca at 21 dpi. We therefore suggest that competition occurs between *C. jejuni* strains for cecal colonization between 7 dpi and 21 dpi, as reported in other experiments (Konkel et al., 2007; Coward et al., 2008; Calderón-Gómez et al., 2009). Our findings show that the *in vitro* classification method developed by our group successfully predicted G2008b as a dominant strain during this cecal co-colonization (Thibodeau et al., 2015a), despite a higher inoculum of D2008b. In addition, in room 2, we found variations in strain ratios between birds inoculated with both strains at 21 dpi, as already observed by other authors (Konkel et al., 2007; Coward et al., 2008). For these authors, stochastic effects including bird-to-bird transmission, population crashes, and phenotypic variations would contribute to these variations. Despite lower levels found in the ileum, we saw that D2008b colonization was decreased in the presence of G2008b at 21 dpi. We therefore demonstrated that *C. jejuni* strains compete for establishment in the ileum. Authors have also reported this competition in their dual infection model with two *C. jejuni* reference strains in chickens (Chaloner et al., 2014). For birds from room 1, there might be a dominance effect observable at the last time point in coherence with the differences in the amount of both strains for some birds.

To explore the underlying mechanisms, both strains were co-cultivated *in vitro* in MH broth. After 48 h of culture, the lower gene copies number observed for D2008b in the presence of G2008b suggests that an interaction occurs between strains. We hypothesize that G2008b could secrete antimicrobial factors in the presence of D2008b to stop its growth, which would explain the absorbance increase at *T* = 24 h when strains were grown together. This increase in the optic density could also be due to an increased overall bacterial death of D2008b. Another hypothesis would be based on a different susceptibility of both strains to oxidative stress resulting from bacterial growth (Nennig et al., 2022). Moreover, both strains might not be able to use the same nutrients as the culture media is becoming depleted, therefore giving an advantage to one strain over the other. Similar *in vitro* experiments have been performed with two *C. jejuni* strains competing for cecal colonization in chickens (Konkel et al., 2007). However, the authors observed no significant difference in the growth rate of strains when cultured alone or together. Future work should focus on investigating the nature of interactions that occur between *C. jejuni* strains. Although differences observed between both strains in our *in vitro* co-culture assays are lower than that of our *in vivo* trial, this suggests that other mechanisms could be involved in strain dominance. However, all of these findings remain to be validated with other competitive strains by performing *in vitro* assays.

Additionally, we observed that a 4 log_10_ increase in the inoculum of D2008b led to its greater dissemination to the liver, likewise reported in the literature (Awad et al., 2020). Moreover, we also noted that G2008b appears to facilitate and accelerate the hepatic dissemination of mainly D2008b, and this was clearly observed when a low dose of D2008b – insufficient on its own to cause intestinal colonization – was used. Recently, similar observations were reported in female turkeys co-inoculated with *C. jejuni* and *C. coli* (Rzeznitzeck et al., 2022). For the first time, we therefore demonstrate that an inoculation with two *C. jejuni* strains would increase liver contamination. Our findings could explain why the majority of studies conducted with a single *C. jejuni* strain show a much lower rate of liver contamination than is found inside retail chicken livers (Knudsen et al., 2006; Pielsticker et al., 2016). Based on these new data, the hepatic spread variability of isolates inoculated separately (Knudsen et al., 2006; Jennings et al., 2011; Pielsticker et al., 2016) would illustrate the need for additional *C. jejuni* strains to improve the hepatic dissemination of a specific strain. In the current study, the dominance of D2008b inside the liver confirms that *C. jejuni* strains have different abilities to invade the internal organs of broiler chickens (Young et al., 1999; Chaloner et al., 2014; Pielsticker et al., 2016). Moreover, in some birds inoculated with both strains, a dominance of D2008b in the liver was associated with a dominance of G2008b in intestinal content. Similar observations have already been reported in a recent study conducted by at the slaughterhouse, showing the isolation of distinct *C. jejuni* strains from the caeca and liver of the same chickens (Berrang et al., 2019). It therefore seems that intestinal colonization and extraintestinal spread involve different mechanisms. In addition, the transcript level decrease of *JAM2*, *CLDN5* and *CLDN10* at 7 dpi observed for some chicken groups inoculated with both strains in both rooms and having livers contaminated by *C. jejuni* suggest that the presence of *C. jejuni* could alter the intestinal epithelial barrier function and enhance the intestinal paracellular permeability, likewise reported in the literature (Lamb-Rosteski et al., 2008; Awad et al., 2020; von Buchholz et al., 2022), leading to extraintestinal spread to internal organs through the bloodstream because of a leaky gut, likely for specific strains only. In view of our results, we suggest that this hypothetical mechanism, leading to the hepatic spread of D2008b, would be enhanced in the presence of G2008b. However, the reason why G2008b did not seem to benefit from this leaky gut by entering the liver remains to be determined. Moreover at 21 dpi, the host response to the hepatic dissemination, like a tissue repair, could explain the increased transcript level of *ZO1*, *JAM2*, *OCLN* and *CLDN10* for the groups of birds having livers contaminated by *C. jejuni*. This hypothesis could thus explain the transient extraintestinal dissemination of *C. jejuni* to the liver. Future research should thoroughly study genotypic and phenotypic characteristics of *C. jejuni* strains able to invade the liver and study the underlying mechanisms of this hepatic dissemination during a multi-strain colonization in broiler chickens.

In conclusion, we showed that the *in vitro* classification developed by our group successfully predicted the competition between D2008b and G2008b, with a dominance of G2008b in the ileum and caeca of birds. For the first time, we observed that commensalism seems to exist between both *C. jejuni* strains, and that this relationship could contribute to intestinal colonization and enhance the hepatic spread of one strain in particular. These results highlight the importance of limiting the introduction of other *C. jejuni* strains by implementing effective biosecurity measures that would help avoid the potential amplification of gut colonization and liver dissemination of certain strains already present on the farm.

However, given the variance of *Campylobacter* colonization in chickens, more animal experimentations should be performed with *C. jejuni* strains in order to confirm the hypotheses of the current study since the lack of replicates in the present study is a clear limitation. For instance, it would be interesting to vary the strains used for inoculation and to sequence them to be able to increase the number of strains for comparative genomics to narrow down the list of genes that might be involved in the differential spread of the strains to the liver. Additionally, using different dose for inoculation and following the animals every day instead of every week would help to better understand what is going on when multiple strains of *C. jejuni* colonize the chicken at the same time. One last limitation of our study is that the number of copy numbers of genes found in each strain was not validated, results that could slightly modify the qPCR results. Future studies should focus on the underlying mechanisms of the commensalism and competition between *C. jejuni* strains during multi-strain colonization in chickens. Such as analysis of bird immune response and intestinal microbiota as an improved understanding of the colonization dynamics between *C. jejuni* strains, alone and together, should be considered in order to develop adequate on-farm control strategies.

## Data availability statement

The original contributions presented in the study are included in the article/Supplementary material, further inquiries can be directed to the corresponding authors.

## Ethics statement

The animal study was reviewed and approved by Comité d’Éthique sur l’Utilisation des Animaux (CÉUA) of the Faculté de Médecine Vétérinaire of the Université de Montréal (certificate number: 19-Rech-2039).

## Author contributions

SC, PF, and AT conceived and designed the experiments. WT provided essential scientific and technical advice. SC, M-LG, WT, and AT actively participated in conducting the *in vivo* experimentations, including bird inoculations, necropsies, and sample processing. SC performed all the experiments in the laboratory. SC and AT analyzed data and wrote the manuscript. M-LG, WT, and PF revised the manuscript. All authors contributed to the article and approved the submitted version.

## Funding

The current study was financially supported by the Natural Sciences and Engineering Research Council of Canada (NSERC grant no. RDCPJ 520873-17), the Consortium de recherche et innovations en bioprocédés industriels au Québec, the Swine and Poultry Infectious Diseases Research Center (CRIPA), The Groupe de Recherche sur les maladies infectieuses en production animale (GREMIP) and the Fonds du Centenaire of the Faculté de Médecine Vétérinaire of the Université de Montréal.

## Conflict of interest

The authors declare that the research was conducted in the absence of any commercial or financial relationships that could be construed as a potential conflict of interest.

## Publisher’s note

All claims expressed in this article are solely those of the authors and do not necessarily represent those of their affiliated organizations, or those of the publisher, the editors and the reviewers. Any product that may be evaluated in this article, or claim that may be made by its manufacturer, is not guaranteed or endorsed by the publisher.
