# Supplementary material for: Observations supporting hypothetical commensalism and competition between two Campylobacter jejuni strains colonizing the broiler chicken gut
Source: Front Microbiol. 2023 Jan 26;13:1071175. doi: 10.3389/fmicb.2022.1071175 (PMC9937062; doi:10.3389/fmicb.2022.1071175)
Supplement: Supplementary file 2 [file Data_Sheet_1.PDF]

Supplementary Figure

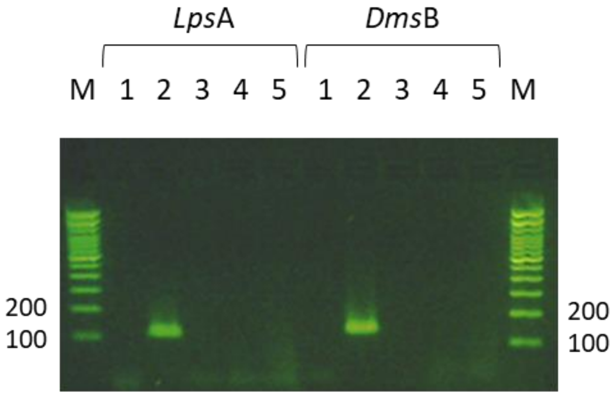

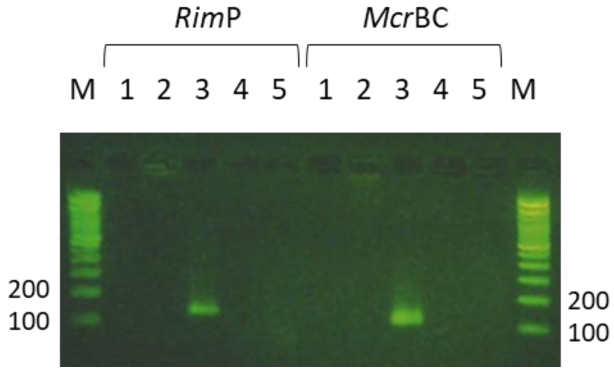

Supplementary Figure S1. Evaluation of *C. jejuni* strain specificity for *Lps*A, *Dms*B, *Rim*P and *Mcr*BC genes by conventional PCR, described in Materials and Methods section. The PCR products were analyzed by electrophoresis on a 2% agarose gel. M = 100-bp ladder; lane 1 = negative control; lane 2 = DNA from G2008b; lane 3 = DNA from D2008b; lane 4 = DNA from caecal content of uncolonized bird; lane 5 = DNA from ileal content of uncolonized bird.
