# Supplementary material for: Observations supporting hypothetical commensalism and competition between two Campylobacter jejuni strains colonizing the broiler chicken gut
Source: Front Microbiol. 2023 Jan 26;13:1071175. doi: 10.3389/fmicb.2022.1071175 (PMC9937062; doi:10.3389/fmicb.2022.1071175)
Supplement: Supplementary file 3 [file Data_Sheet_2.PDF]

Supplementary Table

Supplementary Table S1. Formulation of diets.

Ingredients (%)	Starter phase (0-21 d)[a]	Grower phase (21-35 d)
Corn	31.45	34.66
Soybean meal 48%	25.00	17.70
Wheat	31.24	34.30
Animal fat	2.70	4.40
Corn grains	5.00	5.00
NaCl	0.27	0.27
Limestone	2.13	1.75
Phosphorus	0.99	1.02
L-threonine	0.09	0.04
L-tryptophane	0.03	0.02
L-lysine-HCl	0.31	0.30
DL-methionine	0.30	0.24
L-valine	0.26	0.07
Choline chloride 60%	0.08	0.08
Premix[b]	0.15	0.15

Total	100.00	100.00

[a]The diet phase was changed just after the 7-dpi necropsy.

[b]Premix provided the following nutrients per kg of diet: vitamin A, 12,600 IU; vitamin D3, 3,000 IU; vitamin E, 49.5 IU; vitamin B12, 15 µg; biotin, 201 µg; menadione, 2.55 mg; thiamin, 3.6 mg; riboflavin, 6 mg; pantothenic acid, 14.1 mg; pyridoxine, 3.6 mg; niacin, 52.5 mg; folic acid, 1.5 mg; Fe, 49.5 mg; Cu, 15 mg; Mn, 100.5 mg; Se, 300 µg.
